# Single Mini-incisional Blepharoplasty with the Orbicularis-Orbital Septum Fixation Technique for the Creation of a Natural Double Eyelid

**DOI:** 10.1007/s00266-024-03878-6

**Published:** 2024-02-28

**Authors:** Bo Chen, Li Ma, Huijie Qi, Lizhou Luo

**Affiliations:** 1 Plastic Surgery Department, China Japan Friendship Hospital, No. 2 Yinghuadong Street, Chaoyang District, Beijing, 100029 China; 2 Anesthesiology Department, China Japan Friendship Hospital, Beijing, China

**Keywords:** Blepharoplasty, Double eyelids, Single mini-incision, Orbicularis-orbital septum fixation

## Abstract

**Background:**

Mini-incisional double-eyelid blepharoplasty has increasingly gained popularity in oriental populations. Moreover, many related studies have made a detailed technical introduction to this topic. This study aims to introduce a simple single mini-incisional blepharoplasty procedure with orbicularis-orbital septum fixation technique to create a physiologically natural double eyelid with no visible scar and a short postoperative recovery time.

**Methods:**

A single mini-incisional blepharoplasty with orbicularis-orbital septum fixation technique is described in detail and is illustrated with pictures. Patients who underwent this procedure in our department from November 2020 to May 2023 were retrospectively reviewed

**Results:**

This study included 159 patients who underwent a single mini-incisional blepharoplasty with the orbicularis-orbital septum fixation technique. Surgery duration ranged from 16 to 42 minutes (mean 27 minutes). The majority of the swelling was reduced in 5 days and completely disappeared in 2 weeks for most patients. The follow-up period ranged from 2 to 31 months. All the double eyelids were natural and dynamic, and the creases were stable with no visible scar. Asymmetries occurred in 1.2% (2 of 159), and crease disappeared in 0.6% (1 of 159) of patients; during the follow-up period, no other complications were reported. Of the 159 patients, 99.4% (158) were satisfied with the surgical outcomes

**Conclusions:**

The single mini-incisional blepharoplasty with the orbicularis-orbital septum fixation technique offers a simple, safe, and effective approach to creating double eyelids. It provides natural, stable, scarless, and rapid recovery results with a low risk of complication.

**Level of Evidence IV:**

This journal requires that authors assign a level of evidence to each article. For a full description of these Evidence-Based Medicine ratings, please refer to the Table of Contents or the online Instructions to Authors www.springer.com/00266.

## Introduction

Double-eyelid blepharoplasty has been the most popular aesthetic procedure among the oriental populations. Moreover, some mini-incisional techniques for double-eyelid surgery have been suggested in creating a stable, scarless, and natural-looking pretarsal crease with a short recovery period [1–6]. In these techniques, the incisions, the debulking method, and the fixation technique vary significantly, leading to different postoperative effects. Here, a simple single mini-incisional blepharoplasty procedure with the orbicularis-orbital septum fixation technique is introduced for creating a physiologically natural, stable, and scarless double eyelid with the advantages of short operative time and fast postoperative recovery.

## Patients and Methods

### Patients

This was a retrospective study. This study included and retrospectively reviewed a total of 159 patients who underwent bilateral single mini-incisional double-eyelid blepharoplasty with orbicularis–orbital septum fixation technique at our department from November 2020 to May 2023. Patients with obviously redundant skin, blepharoptosis, previous incisional upper eyelid surgery, or upper eyelid skin disease were excluded from the study. In this study, the procedures were performed according to the ethical standards of the Declaration of Helsinki and the clinical practice guidelines from the hospital’s ethics committee. Preoperatively, signed informed consent and photo release forms were obtained from all patients after fully informing them of the treatment protocol and potential risks. All medical records including age and sex, time of operation, and follow-up period were reviewed and collected. Postoperative evaluation included the swelling of eyelids, double-eyelid stability, double-eyelid crease appearance, and scar formation. Additionally, photographs before and after the surgery were retrospectively examined. Patients’ satisfaction and postoperative complications were also recorded.

### Surgical Technique

#### Design

**Fig. 1:**
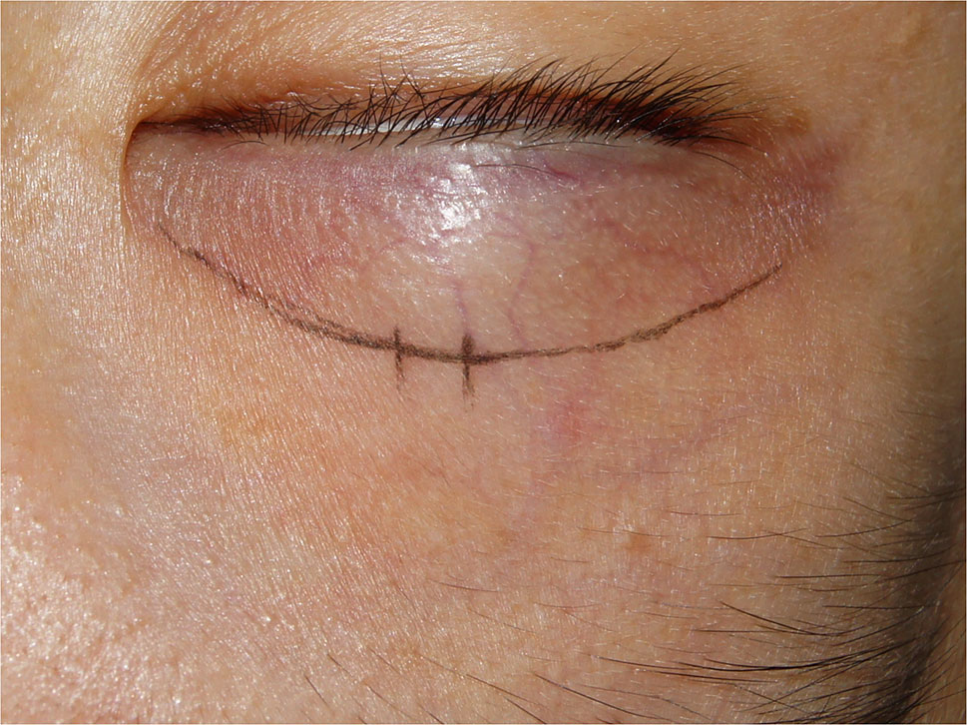
A proper lid crease was designed as the superior border of tarsal plate when the upper lid was slightly retracted upward, and the single mini-incision was marked

Preoperatively, the patient was instructed to remain in an upright sitting position and to keep both eyes at a level to the mirror. The appropriate lid crease was designed according to the superior border of the tarsal plate. While the patient’s eyes were closed, a stainless probe was pressed on the superior border of the tarsal plate, and the upper lid was retracted upward until incipient eyelash eversion was noted. The patient was then instructed to open his/her eyes and look into the mirror while the probe was still in place. The lines could then be moved upward or downward not more than 1 mm until the desired position was confirmed by the patient and a dot would be placed at the central aspect of the proposed crease line. Furthermore, the creases were typically designed 8–11 mm above the superior lid margin when the upper eyelid was slightly retracted upward. The crease line was marked at the supine position, the single mini-incision about 3–5 mm long was clearly marked at the most cephalic part of the crease line and usually right above the pupil. To ensure bilateral symmetry, the lid crease and the single small incision were confirmed several times before surgery (Fig. 1).

### Operative Procedure

**Fig. 2:**
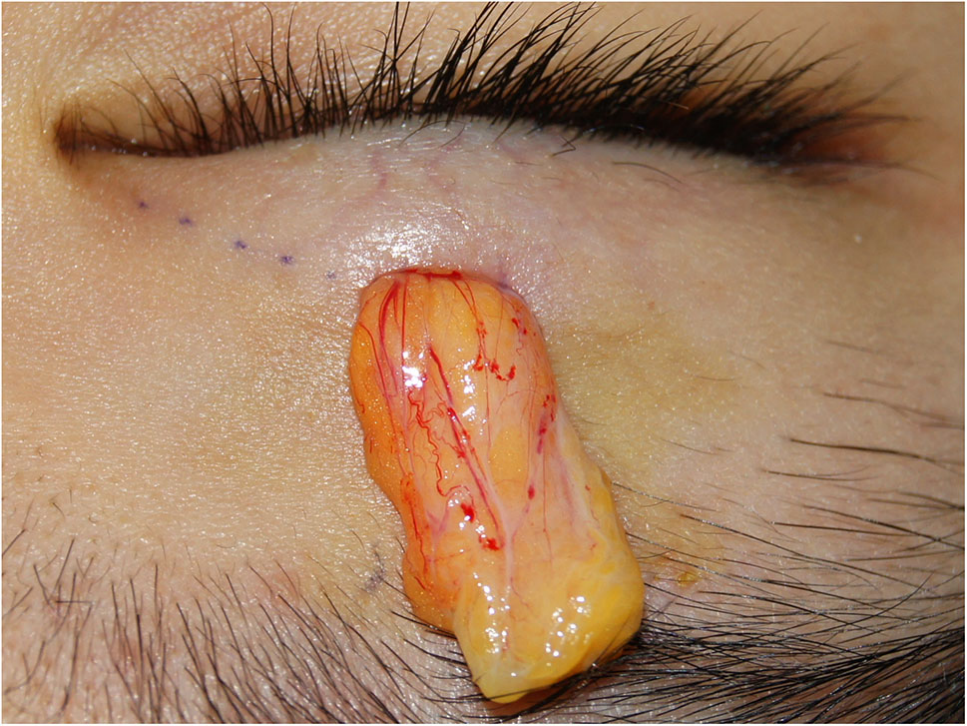
The central preaponeurotic fat was exposed and removed

Local anesthesia was achieved by injecting a small amount of 1% lidocaine and 0.5% bupivacaine with 1:100,000 epinephrine subcutaneously below the marked small incision, typically less than 0.1 ml. The single mini-incision was made using No.11 blade. Through the small incision, the orbicularis oculi muscle and the pretarsal tissue were dissected using a small ophthalmic scissor in a spreading manner, and then the orbital septum and the tarsal plate were exposed. The anterior orbital septum was cut open inwardly and outwardly at the position of about 2 mm above the superior border of the tarsal plate; then, the central preaponeurotic fat and levator aponeurosis were explicitly exposed. In patients with puffy eyelids, the preaponeurotic fat was selectively removed (Fig. 2). The levator aponeurosis was confirmed by picking up the tissue with forceps and pulling it anteriorly and inferiorly; then, the patient was asked to open his/her eye. If the patient had difficulty opening the eye, it means the picked-up tissue was the levator aponeurosis.

**Fig. 3:**
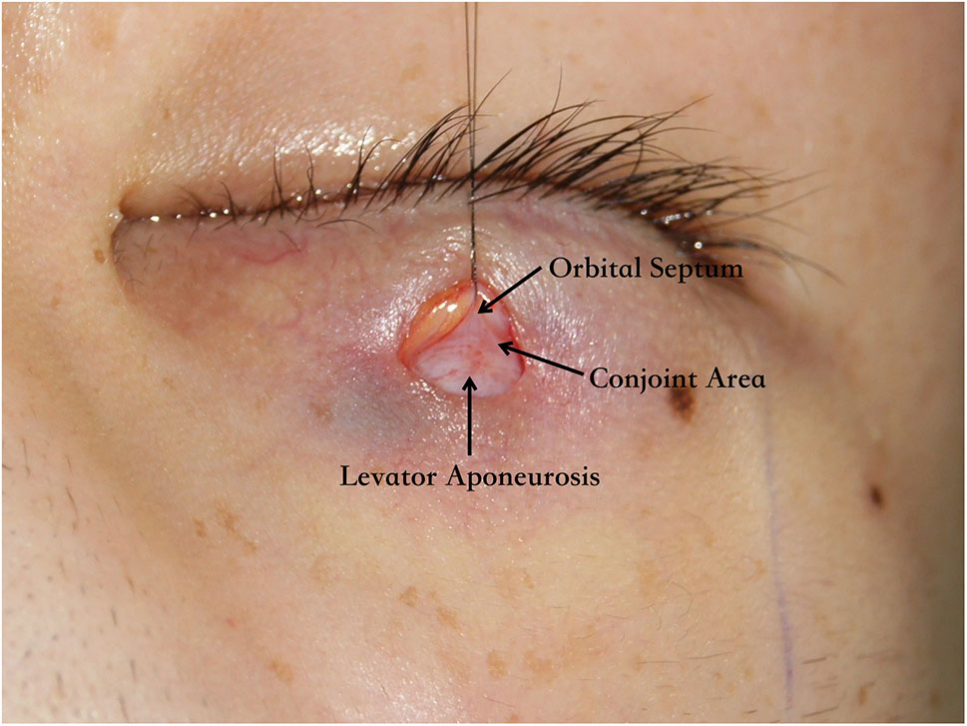
The levator aponeurosis and the anterior orbital septum conjoined at the superior border of tarsal plate

**Fig. 4:**
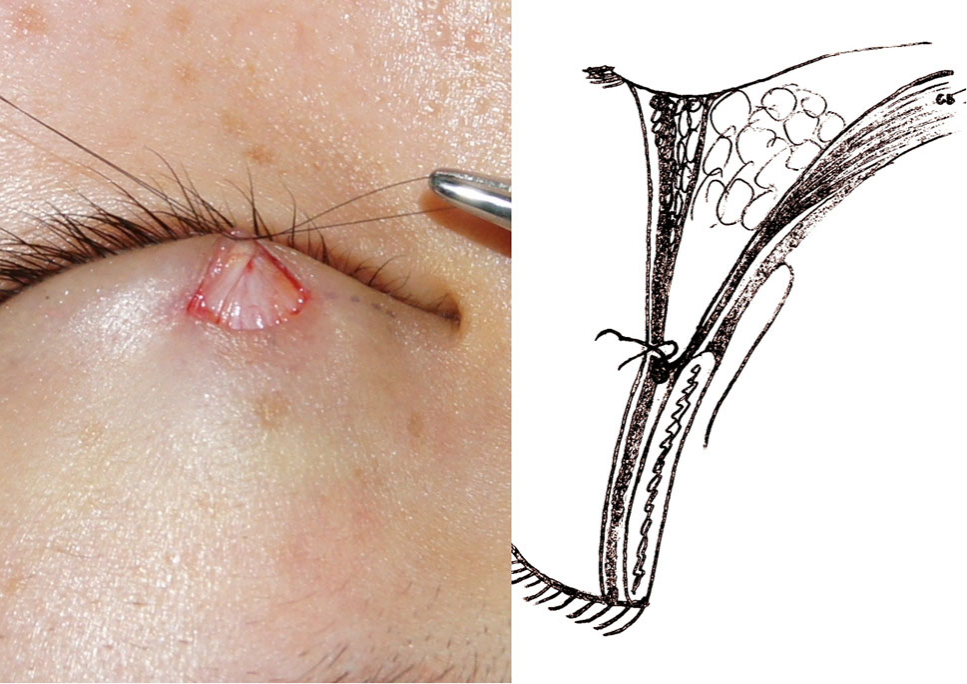
The lower part of the dissected anterior orbital septum was turned downward and was sutured to the inferior orbicularis oculi muscle

**Fig. 5:**
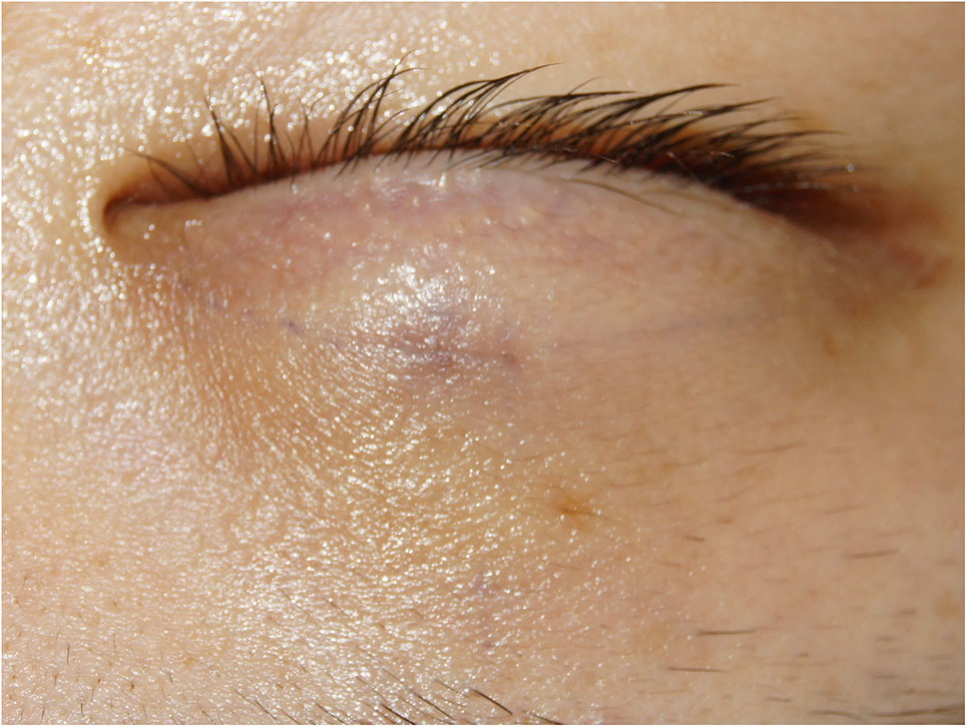
The appearance of the upper eyelid immediately after the single mini-incision was closed using an intradermic suture

The lower part of the dissected anterior orbital septum was turned downward and sutured to the inferior orbicularis muscle with three buried 7-0 nylon sutures which are located in the middle and at the ends of the small incision to simulate a physiologic anatomy of the double eyelid (Figs. 3 and 4). These fixation sutures not only create double-eyelid crease formation at the superior border of the tarsal plate but also avert the eyelashes to correct the lash ptosis. The single mini-incision was closed using an intradermic 7-0 nylon suture (Fig. 5).

### Postoperative Care

Erythromycin eye ointment was applied over the incision immediately after surgery, and the patient was instructed apply ice compress for half an hour before discharge. The patient was advised to apply ice compress as frequently as possible and avoid vigorous exercise for the first 72 hours after surgery. Oral antibiotics were not required, and no suture needed to be removed. Patients could wear makeup 3 days after the surgery and were advised not to rub their upper eyelids for 1 month. Patients were re-examined routinely at 5 days, 2 weeks, and 1, 3, 6, and 12 months after the surgery through the outpatient service or WeChat. All patients were encouraged to return for follow-up if they experienced any problems or complications.

## Results

**Fig. 6:**
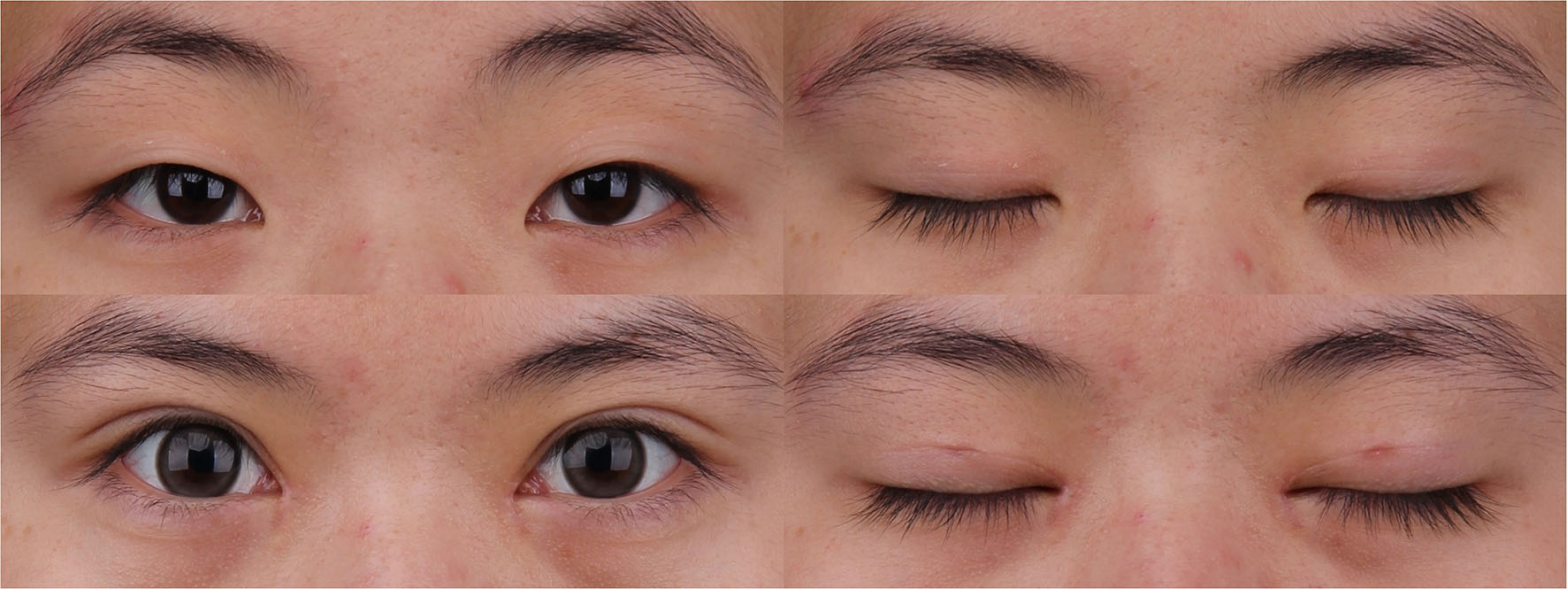
An 18-year-old woman underwent bilateral single mini-incisional blepharoplasty with the orbicularis–orbital septum fixation technique and medial epicanthoplasty simultaneously. Preoperative view of open and closed eyes. Postoperative view of open and closed eyes at 2 weeks

**Fig. 7:**
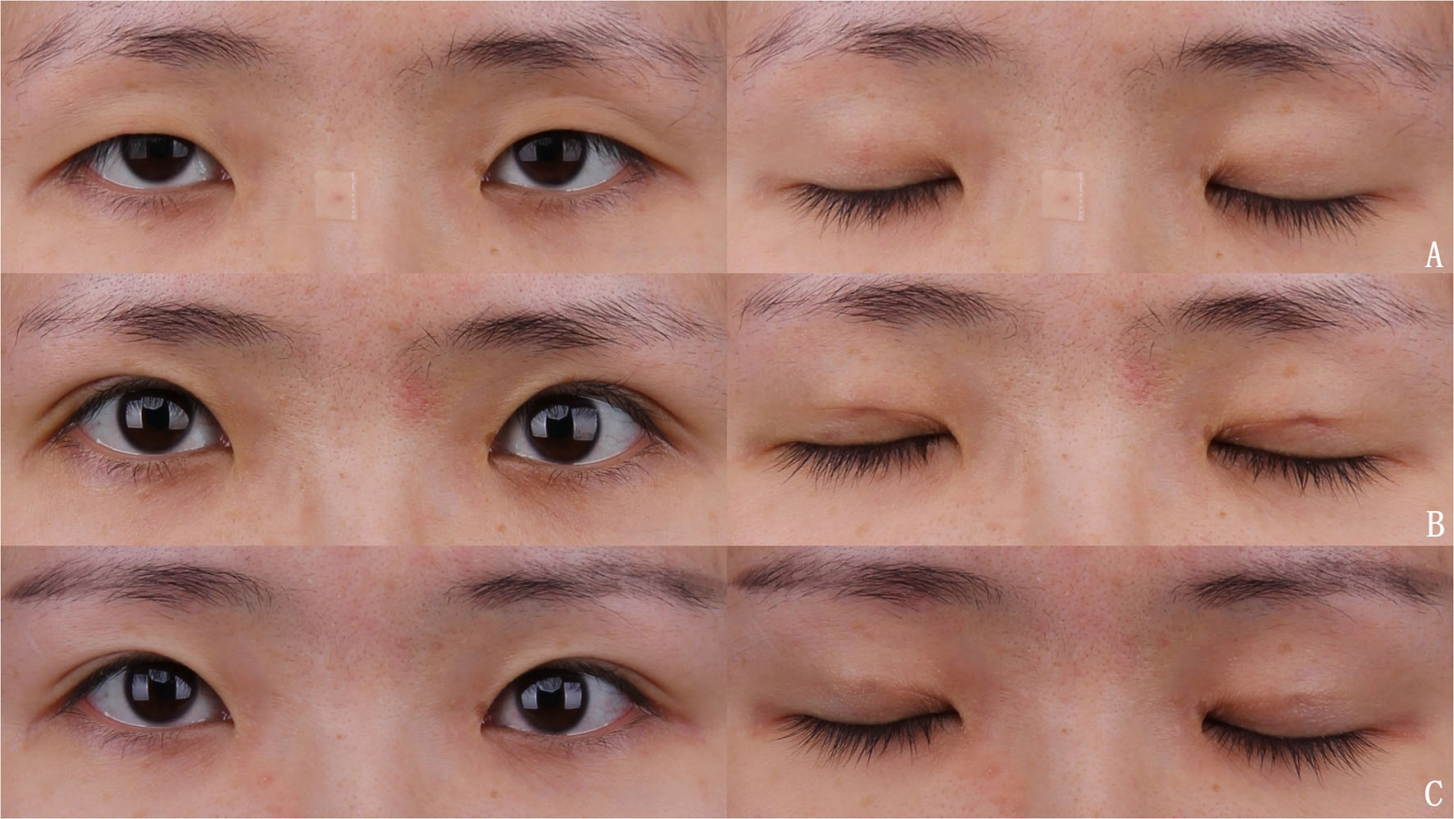
A 27-year-old woman underwent bilateral single mini-incisional blepharoplasty with the orbicularis–orbital septum fixation technique. **A** Preoperative view of open and closed eyes. **B** Postoperative view of open and closed eyes at 1 week. **C** Postoperative view of open and closed eyes at 5 month

This study included 159 patients (135 females, 24 males), with their age ranging from 17 to 41 years and a mean age of 25 years. The duration of operation ranged from 16 to 42 minutes with a mean time of 27 minutes. The majority of the swelling was reduced in 5 days and completely disappeared in 2 weeks for most patients. The follow-up period ranged from 2 to 31 months with a mean period of 16 months. All of the double eyelids were natural and dynamic, and the creases were stable with no visible scar. One patient (0.6%) complained of unilateral fold disappearance at 5 months after the operation, which was then corrected by reoperation. Two patients had asymmetries (1.2%). No other complications including keratopathy, lagophthalmos, ptosis, infection, multiple folds, depression, or persistent swelling were reported during the follow-up period. Of the total patients, 99.4% (158 of 159) were satisfied with the postoperative appearance of the eyelids (Figs. 6 and 7).

## Discussion

The goal of a double-eyelid blepharoplasty is to create a stable and natural-looking pretarsal crease with a short recovery period. For such a purpose, many mini-incisional double-eyelid blepharoplasty techniques have been suggested in recent years [1–10]. In these techniques, the number and length of the incisions, the debulking method, and the fixation and suture technique vary significantly, leading to different postoperative outcomes.

The number of incisions of mini-incisional double-eyelid blepharoplasty ranges from one to four, and the length of each incision ranges from 2 mm to 1 cm, with a total length of all incisions of about 1 cm. The swelling time of these techniques was mostly 2 weeks to 2 month. Our previous study with modified partial incisional technique involved three 2 mm incisions, with the majority of the swelling reduced in 2 weeks and completely disappeared in the first month for most patients [6]. In this study with single mini-incisional and orbicularis-orbital septum fixation technique, the incision was designed to be about 3–5 mm long, the swelling was mild for all patients, and they can go to work or engage in daily communication immediately after the surgery. Moreover, the majority of the swelling was reduced in 5 days and completely disappeared in 2 weeks for most patients. These data suggest that reducing the number and length of incisions can indeed reduce tissue swelling and decrease recovery time.

In most of the previous mini-incisional double-eyelid surgeries, pretarsal orbicular muscle and pretarsal fat tissue were partially or totally removed. The eyelid crease become depressed due to the excessive removal of pretarsal muscle and soft tissue. Additionally, the adhesion between the skin and the tarsus makes it difficult for the pretarsal tissue to move along with eye movements, hence resulting in a stiff appearance. Some experts proposed modified double-eyelid surgery with the removal a strip of orbicularis muscle and pretarsal adipose tissue. After the surgery, the scar contracture and crease depression were noticeable because the skin was directly adhered to the tarsal plate, demonstrating that excessive orbicularis muscle removal could cause scaring and depression [2, 3]. Therefore, to achieve a natural supratarsal crease without obvious depression or conspicuous scar, the orbicularis muscle is kept without excision. In this study, no depression or visible scar was observed, suggesting that orbicularis muscle preservation can effectively prevent eyelid crease depression and alleviate scar.

The deep layer of the levator aponeurosis terminates at the superior border of the tarsal plate, while the superficial layer merges with the lower edge of the orbital septum and extends downward, becoming the pretarsal fascia and covering the anterior surface of the tarsal plate. To generate a double eyelid, the levator aponeurosis, the pretarsal fascia, and the tarsal plate can be regarded as a linked dynamic motor system. Moreover, any one of them or these structures as a whole can be the anchor of the pretarsal fixation [11–16]. After surgery, the double-eyelid crease may appear different, depending on the fixation and suture technique. The levator aponeurosis or tarsal plate can be connected to the skin in various direct and indirect ways. Park et al. proposed the orbicularis–levator fixation [11]. Wu et al. proposed the orbicularis–levator–tarsus composite suture technique [13]. Sun et al. proposed the orbicularis–tarsus fixation [17]. Li et al. proposed the orbicularis–orbital septum fixation [18]. These techniques all features adhesion of orbicularis muscle to the deeper structures and skin-to-skin wound closure. They all suggested that crease formation requires only solid fixation of the orbicularis, other than the skin or the dermis, to the deeper structures. To reconstruct the physiological structure of the double eyelid, Yang et al. proposed the orbital septum–levator aponeurosis flap as a “bridge” and fix to the orbicularis and skin [19].

In our technique, the anterior orbital septum was cut opened about 2 mm above the superior border of the tarsal plate, and the anterior orbital septum was then turned downward and sutured to the inferior orbicularis muscle. After this process, the skin of the tarsal plate was connected to the levator aponeurosis indirectly through the downward overturned anterior orbital septum and orbicularis muscle. This process simulates the physiological fiber connection of the double eyelid, making the double eyelid flexible and natural looking. Additionally, the three buried sutures create a permanent connection between the orbicularis muscle and the orbital septum. The fixation was reliable and will not be interrupted by muscle contraction, and it can ensure the stability and durability of the double-eyelid crease and reduce the risk of fold disappearance. In this study, only one patient (0.6%) complained of unilateral fold disappearance, showing that orbicularis–orbital septum fixation can ensure the stability and durability of the double-eyelid crease.

The keys of this technique include the following: single mini-incision to achieve fast recovery, preservation of the orbicularis muscle to prevent scaring and depression formation, connecting the skin to the levator aponeurosis indirectly through the downward overturned anterior orbital septum and orbicularis muscle to simulate the physiological structure of the double eyelid, and reliable fixation to prevent crease disappearance.

This technique also has several disadvantages: It’s not suitable to patients with excessive skin or poor levator force, who have a possibility of hidden crease appearance or ptosis exacerbation. It also requires a more detailed knowledge regarding the upper eyelid anatomy to identify the orbital septum, levator aponeurosis, and the tarsal plate. This study also has limitations which includes its suboptimal study design and small sample size. The quantified criteria for preoperative and postoperative evaluation and indication for this technique are yet to be set. Recruiting a larger sample size and evaluating the results in a larger time scale will still take some years or more.

## Conclusions

The single mini-incisional blepharoplasty with orbicularis-orbital septum fixation technique offers a simple, safe, and effective approach to create double eyelid for selected patients. It provides natural, stable, scarless, and rapid recovery result with a low risk of complication in a long follow-up period.

## References

[1] Bi YL, Zhou Q, Hu XS, Xu W (2011). Small-incision orbicularis-levator fixation technique: a modified double-eyelid blepharoplasty for treating trichiasis in young Asian patients. J Plast Reconstr Aesthet Surg.

[2] Zhang MY, Yang H, Li CY, Du FY, Huang XJ, Tan WQ (2012). Removal of a large amount of pretarsal tissue through three mini incisions in the construction of a double eyelid. Aesth Plast Surg..

[3] Jinghe Z, Huifang X, Lihong W, Shisheng C, Xiling F (2014). Three mini-incision double-eyelid blepharoplasty. Ann Plast Surg.

[4] Chen B, Song H, Gao Q, Xu M, Wang J, Wang F, Chen S, Wu J, Li H (2017). Measuring satisfaction with appearance: validation of the FACE-Q scales for double-eyelid blepharoplasty with minor incision in young Asians-retrospective study of 200 cases. J Plast Reconstr Aesthet Surg.

[5] Ge M, Pan S, Ni F, Bie Z, Yu D, Song N (2021). Mini-incision blepharoplasty with pretarsal fasciectomy for double-eyelid surgery. Aesthet Plast Surg..

[6] Chen Bo, Ma Li (2023). Small-incision, mini-dissection, orbicularis-preservation, and orbicularis-levator aponeurosis fixation technique: a modified partial-incision double-eyelid blepharoplasty. J Plast Reconstr Aesthet Surg.

[7] Lam SM, Kim YK (2003). Partial-incision technique for creation of the double eyelid. Aesthet Surg J.

[8] Chuangsuwanich A (2006). Short incisional double-eyelid blepharoplasty for Asian patients. Aesthet Surg J.

[9] Zhang YS, Zhou Q, Niu GZ (2019). Individualized small incision orbicularis-levator fixation blepharoplasty for unilateral single-eyelid Asians. J Plast Reconstr Aesthet Surg.

[10] Shen X (2021). Modified double-eyelid blepharoplasty with the combined partial- and minimal-incision method. J Cosmet Dermatol.

[11] Park JI (1999). Orbicularis-levator fixation in double-eyelid operation. Arch Facial Plast Surg.

[12] Kim HS, Hwang K, Kim CK, Kim KK (2013). Double-eyelid surgery using septoaponeurosis junctional thickening results in dynamic fold in asians. Plast Reconstr Surg Glob Open.

[13] Wu LW, Ye Z, Xu Y, Yu J, Wu Y (2015). Orbicularis–levator–tarsus composite suture technique in double-eyelid operation. J Plast Reconstr Aesthet Surg.

[14] Zhou X, Wang H (2019). Orbicularis-white line fixation in asian blepharoplasty: kiss technique. Aesthetic Plast Surg.

[15] Pan L, Sun Y, Yan S, Shi H, Jin T, Li J, Zhang L, Wu S (2019). A flexible suspension technique of blepharoplasty: clinical application and comparison with traditional technique. Aesthetic Plast Surg.

[16] Wang Y, Cao Y, Xie A (2020). A modified procedure for blepharoplasty: physiological structure reconstruction of upper eyelids. J Craniofac Surg.

[17] Sun W, Wang Y, Song T, Wu D, Li H, Yin N (2018). Orbicularis-tarsus fixation approach in double-eyelid blepharoplasty: a modification of Park’s technique. Aesthet Plast Surg..

[18] Li G, Ding W, Tan J, Zhang B, Chen X, He B (2018). A new method for double-eyelid blepharoplasty using orbital septum. Ann Plast Surg.

[19] Jin R, Shen Y, Yu W, Xia Y, Yuan Z, Ding F, Lu L, Liu F, Sun D, Yang J (2020). Tarsal-fixation with aponeurotic flap linkage in blepharoplasty: bridge technique. Aesthet Surg J.

